# Effect of Welsh Onion on Taste Components and Sensory Characteristics of Porcine Bone Soup

**DOI:** 10.3390/foods10122968

**Published:** 2021-12-02

**Authors:** Li Liang, Chenchen Zhou, Yuyu Zhang, Baoguo Sun

**Affiliations:** Beijing Key Laboratory of Flavor Chemistry, Beijing Technology and Business University, Beijing 100048, China; gcfll@126.com (L.L.); zhouchenchen97@163.com (C.Z.); sunbg@btbu.edu.cn (B.S.)

**Keywords:** welsh onion, porcine bone soup, taste components, sensory characteristics, umami

## Abstract

To investigate the effect of welsh onion on taste components and sensory characteristics in porcine bone soup, the stewing condition was as follows: the material–liquid ratio (m/V) was 1:1, the stewing time was 5.0 h, and the ratio of welsh onion was 2.5%. Then, the content of taste components was measured. The content of free amino acids in porcine bone soup with welsh onion (PWS) was higher than the sum of welsh onion soup (WS) and porcine bone soup (PS); particularly, the umami amino acids increased by 35.73% compared with PS. Significant increases in four organic acids (lactic acid, pyroglutamic acid, citric acid and ascorbic acid), two 5′-nucleotides (5′-AMP and 5′-GMP) and three mineral elements (K, Ca and Mg) were observed in PWS. Compared with PS, the equivalent umami concentration (EUC) value was increased from 79.09 to 106.47 mg MSG/100 g in PWS, which was due to the high content of umami amino acids and the synergistic effect with 5′-nucleotides. The results of the sensory analysis indicated a certain enhancement of umami taste in PWS, and the sweet and salty tastes were also increased with the addition of welsh onion. The correlation analysis was consistent with the variation of the components tested above.

## 1. Introduction

Porcine bone is an economical source of flavorful amino acids, peptides, nucleotides, organic acids and other substances [1,2]. Making soup is a good method of cooking porcine bone, which has the characteristics of balanced umami and thick flavor and is rich in protein and other nutrients [3]. The essential amino acids and trace elements in porcine bone soup occupy an indispensable part of people’s diet, and it is favored by consumers. China is one of the major producers and consumers of livestock and poultry. According to the data reported by the Ministry of Agriculture and Rural Affairs of the People’s Republic of China, the number of pigs slaughtered in China was estimated to be 162.59 million in 2020. The porcine bones account for 10% of the carcass, and the amount of pig by-products is huge. The use of nutrients and bioactive compounds such as amino acids and peptides [4] extracted from the porcine bone soup may achieve the maximum utilization of resources [5].

Welsh onion (*Allium fistulosum* L., Alliaceae) has been a traditional medicine and food plant in China, and it is reported to have anti-oxidative, anti-hypertensive and anti-obesity properties [6,7]. *Allium* vegetables are widely used to flavor foods and improve the flavor in foods [8]. In addition, welsh onion is a classic combination with porcine bone in Chinese traditional cooking. Welsh onion is also used globally as a flavor enhancer that is usually cooked together with meats [9,10]. The utilization of welsh onion plays an important role in the industrial production of soup. Studies have shown that welsh onions are rich in bioactive compounds, including polyphenols and organic acids [8,11,12]. Thus, the addition of welsh onion can not only improve the flavor of the food but also increase the nutritional value through antioxidant and antibacterial effects [8]. With the addition of welsh onion extract, the water holding capacity of chicken rolls was increased and the oxidation of chicken fat was reduced, thereby improving the quality of chicken rolls [13]. Additionally, mixed extracts of onion, ginger and garlic were used to preserve the freshness of stewed porcine, which prolonged the shelf life of stewed porcine [14]. Thus, welsh onion may have an important impact on the taste of porcine bone soup during the stewing process.

Amino acids, nucleotides, organic acids, soluble sugars and metal elements contribute to the taste of food [15,16]. Porcine bones are rich in amino acids, including a variety of taste amino acids. For taste amino acids, most hydrophobic L-amino acids exhibit a bitter taste, such as L-Phe, L-Tyr, L-Trp, L-Leu, L-Val and L-Ile; most D-amino acids are sweet, and Gly and L-Ala also have strong sweetness [17]. Organic acids have their own flavor, and they may also enhance other tastes. A mouse model was applied to study fish broth, and it was found that lactic acid can enhance the contribution of amino acids to the taste of soup, while citric acid and lactic acid can change the taste of divalent salt [18]. Taste nucleotides are mainly 5′-adenosine acid, 5′-inosinic acid and 5′-guanylic acid [19], and the synergy effect of taste nucleotides together with amino acids can increase the sensory quality of foods; for example, sodium glutamate and nucleotides together can enhance the umami taste of chicken soup [16]. Mineral elements not only impart a particular flavor but also cooperate with other taste components to enhance the flavor [18]. At present, there are few studies on the effect of welsh onion on the taste and components of porcine bone soup. The current analysis of porcine bones is mostly focused on cooking techniques and the content of amino acids [3,20], and it still lacks comprehensive studies on the contribution of organic acids, nucleotides and mineral elements to the taste of the porcine bone soup.

In this study, the effect of welsh onions on the composition and sensory characteristics of porcine bone soup was investigated. The varieties of amino acids, organic acids, nucleotides and mineral elements after adding welsh onions were explored, and the correlation between taste components and sensory characteristics was also analyzed.

## 2. Materials and Methods

### 2.1. Materials and Reagents

Porcine thigh-bones (femur) and welsh onions were purchased from Yonghui Supermarket (Beijing, China). L-(+)-Tartaric acid, formic acid, lactic acid, acetic acid, citric acid, succinic acid, L-(+)-ascorbic acid, propionic acid, potassium dihydrogen phosphate dodecahydrate phosphate (all AR grade),and malic acid (BR grade) were obtained from Sinopharm Chemical Reagent Co. (Shanghai, China). Potassium dihydrogen phosphate (KH_2_PO_4_), phosphoric acid (H_3_PO_4_), phenol (C_6_H_5_OH), concentrated sulfuric acid (H_2_SO_4_), hydrochloric acid (HCl), disodium hydrogen phosphate dodecahydrate (Na_2_HPO_4_·12H_2_O) (all AR grade), and concentrated nitric acid (HNO_3_) (GR grade) were purchased from Sinopharm Chemical Reagent Co. (Shanghai, China). A multi-element standard solution (50.0 μg/mL) was purchased from the National Center for Analysis and Testing of Nonferrous Metals and Electronic Materials. Inosine 5′-monophosphate (5′-IMP), adenosine 5′-monophosphate (5′-AMP), guanosine 5′-monophosphate (5′-GMP), and cytidine 5′-monophosphate (5′-CMP) were purchased from Sigma-Aldrich (St. Louis, MO, USA). Durashell AA analytical reagents, including an internal standard solution, were purchased from Tianjin Bona Agel Technology Co., Ltd. (Tianjin, China). Methanol, trifluoroacetic acid (TFA), and acetonitrile (ACN) (all HPLC grade) were purchased from Fisher Scientific (Shanghai, China). Ultrapure water was purchased from Hangzhou Wahaha Group Co., Ltd. (Hangzhou, China). Sulfosalicylic acid (AR grade) was obtained from Biochemical Technology Co., Ltd. (Shanghai, China). Salt was purchased from Zhongyan Yangtze Salinization Co., Ltd. (Beijing, China).

### 2.2. Sample Preparation

The fistular onion stalk was taken out of the welsh onion, cut into 5 ± 0.2 cm pieces and split in the middle. The porcine bones were shaved, split in the middle, and washed several times with water until they were clean. The porcine bones were then boiled and rinsed to remove blood foam. For the optimization of stewing time, bone-to-water ratio and the ratio of welsh onion (Table S1), the umami intensity was ranked by using GB/T 12315-2008 [21] and the least significant difference (LSD) between samples was calculated by the following formula. There is a significant difference if the difference of rank-sum between the two samples was not lower than LSD.

(1) $$\mathrm{LSD}=1.96\sqrt{\frac{j\times p(p+1)}{6}}$$

Here, *p* and *j* are the number of samples and panelists, respectively.

Based on the result in Figure S1, the stewing conditions were optimized: the material–liquid ratio (m/V) was 1:1, the stewing time was 5.0 h, and the ratio of welsh onion was 2.5%. According to the optimized conditions, the sample of welsh onion soup (WS), porcine bone soup (PS) and porcine bone soup with welsh onion (PWS) were prepared in a nutrient soup mode using a DGD40-40DWG electric stew pot (Guangdong Tonze Electric Co., Ltd., Guangdong, China). The soups were then cooled to room temperature, then filtered and centrifuged by a TGL16M centrifuge (Hunan Xiang Yi Laboratory Instrument Development Co., Ltd., Hunan, China) at 10,000 r/min for 10 min to remove impurities. For groups WS, PS and PWS, stewing was repeated three times and the soups were mixed together as one. Samples were then collected from the three groups using the five-point sampling method [22], and the collected sample with a certain quantity was retrieved in triplicate for each analysis. Sensory analysis was performed after sample collection, and other samples were stored in sterilized vials at −20 °C for further analysis.

### 2.3. Free Amino Acid Analysis

The quantitative analysis of amino acids was carried out as described in the literature [23] with some modifications. A 1 mL sample was added to 1 mL 10% (*v*/*v*) 5-sulfosalicylic acid solution and diluted to 25 mL with 0.1 mol/L HCl. Then, it was mixed with an internal standard solution and filtrated through a 0.22 µm nylon filter membrane before HPLC analysis. The chromatography Durashell AA (4.6 mm × 150 mm, i.d. 3 µm) a column (Agela Technologies, Tianjin, China) and the Durashell AA analysis kit (Agela Technologies, Tianjin, China) were used to analyze the free amino acid in WS, PS and PWS. Mobile phase A (4.50 mg/mL Na_2_HPO_4_ and 4.75 mg/mL Na_2_B_4_O_7_, pH = 8.2, water solution) and mobile phase B (methanol: acetonitrile: water = 9:9:2, *v*/*v*/*v*) were used for the elution solvent. A 1260 Agilent HPLC (Agilent Technologies Inc., Santa Clara, CA, USA) coupled with a diode array detector (DAD) was used for free amino acid analysis. The gradient elution method was 6–10% B for 0–6 min, 10% B for 6–8 min and 10–16% B for 8–10 min; the flow rate of the mobile phase was 1.6 mL/min; the column temperature was 45 °C; and the detection wavelengths were 338 and 262 nm.

### 2.4. Organic Acid Analysis

The samples (2 mL) were filtered through a 0.22 µm nylon filter membrane before analysis. Venusil XBP C18 (4.6 mm × 250 mm, 5 μm) was used to analyze the organic acids. Identification and quantification of the organic acids were conducted according to the literature [24]. The mobile phase comprised buffer salt I (0.01 mol/L KH_2_PO_4_, pH = 2.8) and methanol (5:95, *v*/*v*), which were used for equal gradient elution at a flow rate of 1 mL/min, with an injection volume of 20 μL. A Thermo U3000 UPLC system (Thermo Fisher Scientific, Wilmington, DE, USA) was used to analyze the organic acids. The organic acids were detected at a wavelength of 205 nm; the column temperature was 25 °C. The external standard method was used for quantitative analysis; we set the mixed standard concentrations of organic acid to 10.00, 5.00, 3.00, 1.00, 0.80, 0.50, 0.20 and 0.08 μg/mL. The test under the above conditions and the peak area of the obtained chromatogram were plotted against the concentration to draw the standard curve of organic acid.

### 2.5. 5′-Nucleotide Assay and Equivalent Umami Concentration

The pretreatment method of the sample was conducted based on the method described in the literature [25]. The instrument, detector, column, column temperature, and mobile phase elution gradient were similar to those used for the analysis of the organic acids. The nucleotides were detected at 254 nm. The mobile phase comprised methanol-buffer salt II (5:95, *v*/*v*) at a flow rate of 1 mL/min. The mixed nucleotides (5′-AMP, 5′-GMP, 5′-IMP and 5′-CMP) were prepared into 0.1 mg/mL calibration standard solution with ultrapure water, then diluted into seven gradients. Each 5′-nucleotide was quantified according to the calibration curve of the standard 5′-nucleotide.

Since disodium nucleotide and monosodium glutamate have a synergistic effect, the equivalent umami concentration (EUC) formula was used to convert the umami intensity presented by the mixed solution into the equivalent umami concentration of monosodium glutamate (MSG).

(2) $$\mathrm{EUC}=\sum a_{i}b_{i}+1218\left(\sum a_{i}b_{i}\right)\left(\sum a_{j}b_{j}\right)$$

Here, EUC is MSG equivalent (g MSG/100 g), *a_i_* and *a_j_* are the concentration of umami amino acids and nucleotides (g/100 g), respectively; *b_i_* is the relative umami concentration (RUC) for each umami amino acid versus MSG (Glu, 1 and Asp, 0.077); *b_j_* is the RUC for each umami 5′-nucleotide versus 5′-IMP (5′-IMP, 1; 5′-GMP, 2.3 and 5′-AMP, 0.18), and 1218 is a synergistic constant based on the concentration of g/100 g.

### 2.6. Mineral Element Analysis

Mineral elements were measured according to a previously described method [20,26]. For samples WS, PS and PWS, 5 mL of the sample was accurately transferred into the digestion tank, 6 mL of concentrated HNO_3_ was added, and the sample was pre-digested for 30 min at 120 °C. It was then taken out and cooled to room temperature 25 °C. Microwave digestion was carried out in a JUPITER-B microwave digestion workstation (Shanghai Sineo Microwave Chemistry Technology Co., Ltd., Shanghai, China) according to the procedure detailed in Table 1. The acid was then removed at 160 °C until the remaining sample was about 1 mL. The constant volume was then taken out to 25 mL. The blank was treated as above.

The multi-element standard solution was used to qualitatively and quantitatively analyze the metal elements in the samples by the standard curve method. The measurement was carried out using ICPE-9800 (Shimadzu, Hong Kong, China) according to the measurement conditions shown in Table 2.

### 2.7. Taste Traits by Electronic Tongue Analysis

The taste traits of WS, PS and PWS were evaluated by an Electronic tongue (INSENT SA402B, Tokyo, Japan) following the reported method [27] with some modifications. The electronic tongue was composed of six taste sensors for sourness (CA0), bitterness (C00), astringency (AE1), umami (AAE), saltiness (CT0) and sweetness (GL1). The SA-402B taste analysis system uses artificial lipid membrane sensing technology similar to the working principle of taste bud cells. The artificial lipid membrane produces changes in the membrane potential through electrostatic or hydrophobic interaction with taste-bearing substances. The electric potential is used as the output of the sensor, and the signal is transmitted to the computer for analysis to recognize the taste intensity and taste characteristics. Before analysis, the sensor was pretreated in a reference solution (30 mM KCl solution containing 0.3 mM tartaric acid) for 24 h, and then the WS, PS and PWS samples (50 mL) were filtered and degreased for measurement. The program was set with a sample taste collection time of 30 s, an aftertaste collection time of 30 s, and a cleaning time of 300 s. The test was conducted at a room temperature of 25 °C.

### 2.8. Sensory Evaluation

All recruited panelists were informed of the detailed steps and aims of the sensory evaluation. Then they were provided with written informed consent. According to the training methods in the literature [28], 10 panelists (5 men and 5 women aged between 22 and 30, healthy and non-smoking with no taste/odor disorders) were trained by the ranking test for 3 weeks. The taste solutions including glucose (50.00 g/L), citric acid (5.00 g/L), sodium chloride (15.00 g/L), sodium glutamate (20.00 g/L), and quinine (6.00 mg/L), were respectively diluted in 2-fold serials of 1:2, 1:4, 1:6, 1:8, and 1:16. Each of the taste solutions with five different concentrations was presented to the panel, and they were asked to rank the orders according to taste perception intensity. Panelists were requested to participate in the 2-AFC (a two-alternative forced-choice) test according to ISO 5495. Only the panelists with correct answers (100% cut-off point) were selected to participate in further exploration.

According to “GB/T19547-2004” [29], the soup samples (50 mL) were put into a bottle and randomly numbered with three digits. After the training, the qualified panelists were asked to take a sip of the sample and to keep it in the mouth for 10 s, then spit it out. The intensity of each attribute (sourness, sweetness, bitterness, saltiness, umami and astringency) was scored by the panelists according to the degree for sensory evaluation (Table S2). At the interval between samples, panelists were asked to wash their mouth with 50 mL drinkable water to avoid fatigue and carryover effect. The sensory evaluation experiment was performed on different groups at a 1-hour interval.

### 2.9. Statistical Analysis

Statistical analysis was performed using SPSS software (version 19.0, SPSS Inc., Chicago, IL, USA) and Excel (20 10, Microsoft Co., Ltd., Redmond, WA, USA) [30,31]. All statistical analyses with the experimental results are expressed as means ± standard deviation. One-way analysis of variance and Duncan’s multilevel tests were applied for determining significant differences at *p* < 0.05.

## 3. Results and Discussion

### 3.1. Comparison of Free Amino Acids

Free amino acids have a significant contribution to the overall flavor in food [32]. Table 3 shows the content and taste attributes of free amino acids (FAAs) in the WS, PS and PWS samples. Except for the content of glycine and valine, the content of the other 14 FAAs increased after adding welsh onion and porcine bone. For the total of FAAs in WS, PS and PWS, the contents were 79.26, 373.37 and 478.73 mg/L, respectively. Due to the same process conditions, PWS should numerically equal the sum of WS and PS, but the FFAs content of PWS increased by 4.66% compared to the sum of WS and PS. The addition of welsh onion may promote the release of FAAs, and this phenomenon was also found in chicken soup with ginger [33]. It indicated that spices have effects on free amino acids in meat soups, but the mechanism needs to be further studied. According to the taste attributes, the FAAs in WS accounted for 9.56% of umami amino acids, 30.88% of sweet amino acids, 54.04% of bitter amino acids and 5.52% of tasteless amino acids. In the PS, they accounted for 15.81% of umami amino acids, 27.00% of sweet amino acids, 48.81% bitter amino acids and 8.38% of tasteless amino acids. In the PWS, umami amino acids took 18.89%, the sweet amino acids took 26.55%, the bitter amino acids took 46.51%, and the tasteless amino acids took 8.05%. In summary, compared with the sum of WS and PS, sweet amino acids increased by 1.45% and umami amino acids increased by 35.73% in PWS. With the addition of welsh onion, the content of aspartic acid glutamic in PWS increased significantly. Aspartic acid and glutamic acid are umami amino acids and contribute to the overall taste of food along with other substances. Phenylalanine elicits a bitter taste, but recently it has been discovered to be an important component of the soy sauce savory fractions in addition to glutamate [34]. The results suggested that the addition of welsh onions increased the FAAs in porcine bone soup, especially the umami amino acids, which may promote the taste quality of the porcine bone soup.

### 3.2. Comparison of Organic Acid

The content of organic acids is another important factor in the taste of food. It provides a sour taste, and some organic acids can also act as flavor enhancers [18]. The organic acid contents in the WS, PS and PWS samples are shown in Table 4. Eight organic acids were detected in the three kinds of soup, and the content of total organic acids in PWS was significantly higher than that in PS, indicating that the addition of welsh onions increased the content of organic acids in porcine bone soup. Compared with the sum of WS and PS, the content of lactic acid, pyroglutamic acid, citric acid and ascorbic acid were increased in PWS, indicating that the compatibility of welsh onions and porcine bones promoted the release of lactic acid, pyroglutamic acid, citric acid and ascorbic acid. Tartaric acid, citric acid and succinic acid covered a large proportion of organic acid in PWS; in addition, lactic acid may also have an impact on the flavor of porcine bone soup. The lactic acid in whey permeate has the greatest salty taste enhancement effect in permeate [35]. The influence of organic acids on the umami taste of beef-flavored ramen soup was investigated, and the result showed that citric acid and lactic acid can replace part of MSG and have the effect of enhancing umami taste [36]. The results indicated that the increase of citric acid and lactic acid contributed to the umami taste of porcine bone soup with the addition of welsh onion.

### 3.3. Comparison of 5′-Nucleotide and EUC

The nucleotides, including 5′-IMP, 5′-GMP, 5′-CMP and 5′-AMP, are umami components and contribute to the flavor quality of food. There is also a synergistic effect between nucleotides and amino acids, which plays a key role in enhancing the umami taste [16]. The results of nucleotides content are listed in Table 5. Significant differences in total nucleotides were not observed between PWS and PS. Among four detected nucleotides, the content of 5′-AMP and 5′-GMP in PWS was significantly higher than that in WS and PS. 5′-GMP is a notable flavor substance because of its large proportion and savory meat flavor [37].

The EUC value has usually been used to reflect the umami intensity of samples [38]. To further evaluate the synergistic effect of umami amino acids and nucleotides, the EUC value of the three kinds of soup was analyzed and the results are shown in Table 6. The EUC values of WS, PS and PWS were 4.87, 79.09 and 106.47 mg MSG/100 g, respectively. The EUC value of WS was significantly (*p <* 0.05) lower than that in PS and PWS. The EUC value of PWS was significantly (*p <* 0.05) higher than the sum of WS and PS (83.96 mg MSG/100 g); therefore, the umami taste was significantly promoted with the addition of welsh onion. The reasons for this phenomenon were the high content of FAAs and the synergistic effect between umami amino acids and nucleotides.

### 3.4. Comparison of Mineral Elements

In the present study, five mineral elements (Al, Ca, Mg, K and Na) were detected in different samples, and the results are shown in Table 6. Elements K and Na act as regulators of cell osmotic pressure and acid–base balance, which is important to human health. In samples of PS and PWS, the contents of K and Na were significantly higher than other mineral elements; thus, elements K and Na were the major mineral elements. Compared with PS, the addition of welsh onion increased the content of K, Ca and Mg in PWS. It could be that the mineral elements in the welsh onion were released into the soup after the stewing. However, the Ca, K, Mg, Al and Na elements in PWS were lower than the sum of WS and PS. In a soup system, mineral elements may combine with fatty acids to generate fatty acid salts and form oil in water emulsion [16], leading to the above results. Ca was one of the major elements in WS, and the content was about 118.18 mg/L. Furthermore, the content of Ca in PWS (129.00 mg/L) was significantly higher than that in PS (63.25 mg/L). The pseudostem of welsh onions is high in element Ca [39]; thus, the addition of welsh onions substantially increased the Ca content and improved the nutritional quality in porcine bone soup. In addition, the content of Na in PWS (706.25 mg/L) was significantly lower than that in PS (758.75 mg/L), which indicates that the addition of welsh onion may inhibit the release of Na from porcine bones.

### 3.5. Sensory Evaluation Results

**Figure 1:**
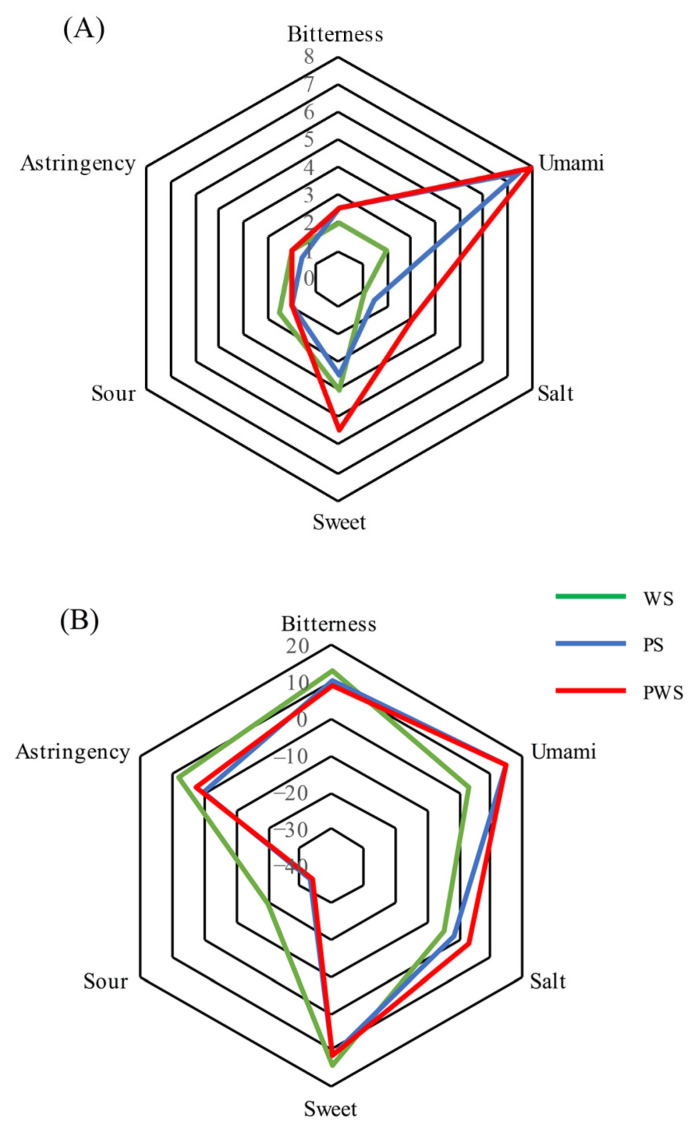
WS, PS and PWS sensory evaluation scores (**A**) and electronic tongue score map (**B**).

The sensory evaluation results of WS, PS and PWS are shown in Figure 1A. It can be seen that WS was mainly tasted as bitter, astringent, and sweet, and the taste was relatively weak. Various alkaloids, polyphenols and flavonoids have been detected in aqueous extracts of welsh onion [8], by which bitterness and astringency might be introduced [40,41]. PS was dominated by umami among the six tastes. After the addition of welsh onions, the taste profile of porcine bone soup was basically the same. But the saltiness and sweetness were significantly higher than in PS, and umami taste was also improved slightly. This result is consistent with the variation of the amino acid (Table 3), in which the bitter amino acid decreased and the umami amino acid increased after the addition of welsh onions. It was concluded that welsh onions contribute to endow porcine bone soup with better taste. The electronic tongue was also applied to analyze the taste quality of WS, PS and PWS, and the results are shown in Figure 1B. In general, valid responses were observed from the results of the electronic tongue, except for sourness. When the sourness response of the electronic tongue is less than −13, it means that the sample has no sour taste compared with the reference solution and the electronic tongue failed to respond to the sour taste of the three soups. As shown in Figure 1B, the bitterness and astringency in PWS were reduced compared with WS. When compared with PS, the salty tastes were increased with the addition of welsh onion, which was consistent with the results of sensory evaluation.

### 3.6. Correlation Analysis Results

**Figure 2:**
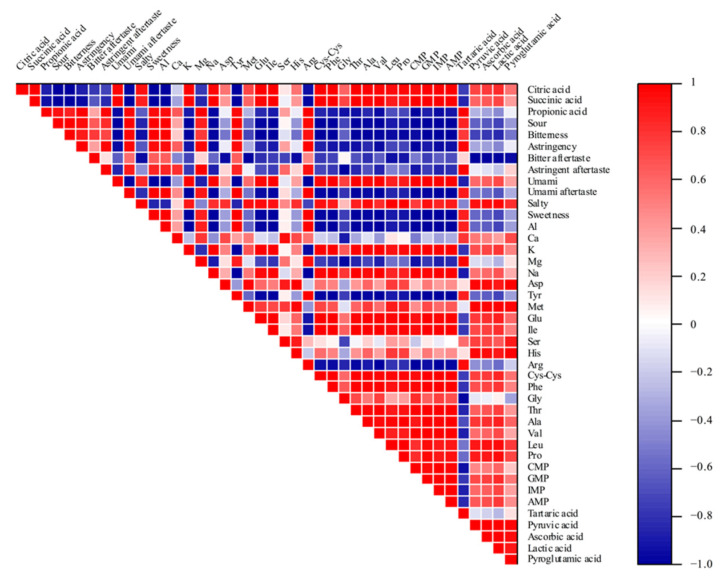
Correlation analysis of sensory properties and taste components.

Porcine bone soup is a complex system and its sensory properties are related to the composition and content of the substances, which may have a particular effect on the taste. To further investigate the effect of the components on taste quality, correlation analysis was carried out, and the results are shown in Figure 2. The results of the correlation analysis indicated that the variation of components has a powerful impact on sensory properties in the system of porcine bone soup. The content of tartaric acid, Arg, Ser, Tyr, Mg, Ca and Al was positively correlated with sourness, bitterness, astringency and sweetness, and additionally, they were negatively correlated with umami and saltiness. Specifically, the umami taste had a highly positive correlation with citric acid, succinic acid, lactic acid, K, Na, Cys, umami amino acids (Glu and Asp), bitter amino acids (Ile, Phe, Val and Leu), sweet amino acids (Gly, Pro, Ala and Thr) and nucleotides (5′-IMP, 5′-GMP, 5′-CMP and 5′-AMP). This is consistent with the analysis of taste components above. After the addition of welsh onion, the umami taste was significantly promoted with the increase of positively correlated components, especially the content of umami amino acids, most sweet amino acids and some bitter amino acids. Moreover, there was a positive correlation between umami and saltiness, and the components that contributed to umami taste and salty taste were similar. The salty taste is closely related to the umami taste [42]; when umami components exist, saltiness could also be identified because of the complementary effect [43].

## 4. Conclusions

In summary, the addition of welsh onion can improve the sensory quality of porcine bone soup by affecting the related components. Porcine bone soups were prepared under these conditions: the material–liquid ratio (m/V) was 1:1, the stewing time was 5.0 h, and the ratio of welsh onion added was 2.5% (accounting for bone mass). Variations of amino acids, organic acids, nucleotides, and mineral elements were detected in bone soup with or without welsh onion. The results suggested that the addition of welsh onions increased the free amino acids in porcine bone soup, especially the umami amino acids (increased by 35.73%), which contribute to promote the taste quality of porcine bone soup. Moreover, the release of lactic acid, pyroglutamic acid, citric acid, and ascorbic acid was significantly promoted with the addition of welsh onion. Compared with PS, the EUC value was increased from 7.85 to 9.71 g MSG/100 g in PWS, which was due to the high content of umami amino acids and the synergistic effect with 5′-nucleotides. In porcine bone soup with welsh onion, the enhancement of umami taste was verified by sensory evaluation, and the connection between tastes and certain components was revealed according to a correlation analysis.

## Figures and Tables

**Table 1:** Microwave digestion program.

Step	Temperature/°C	Power/%	Time/min
1	150	1000	10
2	180	1000	15

**Table 2:** Instrument measurement conditions.

Parameter	Value
High frequency power	1.20 kW
Plasma gas	10.00 L/min
Auxiliary gas	0.60 L/min
Carrier gas	0.70 L/min

**Table 3:** The contents and taste attributes of free amino acids (FAA) in three samples.

Taste	FAAs	Content (mg/L)		
		WS	PS	PWS
Umami	Aspartic acid	3.20 ± 0.01 ^b^	1.35 ± 0.02 ^a^	14.16 ± 0.05 ^c^
	Glutamic acid	4.37 ± 0.03 ^a^	57.69 ± 0.06 ^b^	76.25 ± 0.15 ^c^
Sweet	Threonine	nd	4.26 ± 0.01 ^a^	4.46 ± 0.01 ^b^
	Serine	16.12 ± 0.03 ^a^	nd	28.69 ± 0.10 ^b^
	Glycine	nd	36.12 ± 0.12 ^b^	11.37 ± 0.05 ^a^
	Proline	8.36 ± 0.70 ^a^	15.09 ± 0.29 ^b^	20.07 ± 0.52 ^c^
	Alanine	nd	45.33 ± 0.01 ^a^	62.51 ± 0.17 ^b^
Bitter	Histidine	13.57 ± 0.06 ^b^	11.63 ± 0.02 ^a^	25.83 ± 0.10 ^c^
	Tyrosine	3.07 ± 0.00 ^a^	nd	nd
	Valine	nd	46.97 ± 0.69 ^b^	45.83 ± 0.67 ^a^
	Methionine	2.20 ± 0.01 ^a^	2.98 ± 0.01 ^b^	9.44 ± 0.08 ^c^
	Isoleucine	3.65 ± 0.01 ^a^	34.94 ± 0.12 ^b^	41.21 ± 0.55 ^c^
	Phenylalanine	3.16 ± 0.01 ^a^	69.86 ± 1.35 ^b^	79.83 ± 6.47 ^c^
	Argnine	17.18 ± 0.14 ^c^	11.69 ± 0.07 ^b^	12.80 ± 0.04 ^a^
	Leucine	nd	4.12 ± 0.58 ^a^	7.68 ± 0.79 ^b^
-	Cystine	4.38 ± 0.00 ^a^	31.29 ± 0.01 ^b^	38.52 ± 0.04 ^c^
Total		79.26 ± 0.61 ^a^	373.37 ± 3.41 ^b^	478.73 ± 8.46 ^c^

Note: nd means not detected. Different letters in the same row represent significant differences according to Duncan’s test (*p* < 0.05).

**Table 4:** Contents of organic acids in three samples.

Organic Acids	Content (mg/L)		
	WS	PS	PWS
Tartaric acid	2027.37 ± 114.87 ^c^	761.42 ± 24.38 ^a^	1397.24 ± 144.42 ^b^
Pyruvic acid	1.94 ± 0.41 ^a^	3.16 ± 0.35 ^a^	11.60 ± 1.43 ^b^
Ascorbic acid	1.25 ± 0.26 ^a^	6.88 ± 0.23 ^a^	33.47 ± 4.48 ^b^
Lactic acid	165.34 ± 35.62 ^a^	272.60 ± 12.12 ^b^	541.39 ± 39.89 ^c^
Pyroglutamic acid	42.78 ± 4.94 ^a^	30.98 ± 1.93 ^a^	111.21 ± 13.23 ^b^
Citric acid	65.37 ± 6.88 ^a^	3232.18 ± 93.62 ^b^	4009.09 ± 458.60 ^c^
Succinic acid	260.38 ± 81.65 ^a^	3699.27 ± 170.89 ^b^	3793.55 ± 431.01 ^c^
Propionic acid	202.51 ± 33.90 ^c^	3.64 ± 1.72 ^a^	70.39 ± 14.34 ^b^
Total	2787.91 ± 235.09 ^b^	8010.15 ± 368.87 ^a^	9967.97 ± 1354.13 ^c^

Note: Different letters in the same row represent significant differences according to Duncan’s test (*p* < 0.05).

**Table 5:** Contents of nucleotides and equivalent umami concentration (EUC) of the three samples.

Compounds	Content (mg/L)		
	WS	PS	PWS
5’-CMP	17.84 ± 0.19 ^a^	38.34 ± 2.34 ^b^	35.16 ± 0.92 ^b^
5’-GMP	24.09 ± 0.03 ^a^	24.99 ± 0.02 ^b^	25.18 ± 0.06 ^c^
5’-IMP	21.24 ± 0.03 ^a^	42.94 ± 0.34 ^b^	42.99 ± 0.92 ^b^
5’-AMP	9.18 ± 0.01 ^a^	20.50 ± 0.16 ^b^	21.52 ± 0.54 ^c^
Total	72.37 ± 0.23 ^a^	126.80 ± 2.96 ^b^	124.87 ± 2.96 ^b^
EUC (mg MSG/100 g)	4.87 ± 0.054 ^a^	79.09 ± 0.44 ^b^	106.47 ± 1.54 ^c^

Note: Different letters in the same row represent significant differences according to Duncan’s test (*p* < 0.05).

**Table 6:** Mineral contents of the three samples.

Elements	Content (mg/L)		
	WS	PS	PWS
Al	0.05 ± 0.00 ^a^	nd	nd
Ca	118.18 ± 0.46 ^b^	63.25 ± 0.11 ^a^	129.00 ± 0.39 ^c^
K	150.25 ± 0.36 ^a^	936.25 ± 0.82 ^b^	1051.25 ± 1.78 ^c^
Mg	29.00 ± 0.00 ^c^	12.83 ± 0.01 ^a^	20.83 ± 0.00 ^b^
Na	56.43 ± 0.22 ^a^	758.75 ± 2.27 ^c^	706.25 ± 3.49 ^b^

Note: nd means not detected. Different letters in the same row represent significant differences according to Duncan’s test (*p* < 0.05).

## Data Availability

The authors declare that all the data supporting the findings of this study are available within the article.

## Supplementary Materials

The following are available online at https://www.mdpi.com/article/10.3390/foods10122968/s1, Figure S1: Optimization of stewing conditions: (A) Stew time; (B) stew bone–water ratio (m/V); and (C) ratio of welsh onions added (percentage of bone mass) by sensory evaluation, Table S1: Optimization factors for bone soup stewing conditions, Table S2: Degree for sensory evaluation of taste attributes.
