# Supplementary material for: Effect of Welsh Onion on Taste Components and Sensory Characteristics of Porcine Bone Soup
Source: Foods. 2021 Dec 2;10(12):2968. doi: 10.3390/foods10122968 (PMC8701721; doi:10.3390/foods10122968)
Supplement: Supplementary file 1 [file foods-10-02968-s001.zip › foods-1429785-supplementary.pdf]

Table S1. Optimization factors for bone soup stewing conditions.

Optimal conditions		
Time /h	m(bone): V(water) /(g/mL)	Welsh onion / (percentage of bone)
2	1:1.0	1.0%
3	1:1.5	1.5%
4	1:2.0	2.0%
5	1:2.5	2.5%
6	1:3.0	3.0%

Table S2. Degree for sensory evaluation of taste attributes.

Score	Concentration (mg/mL)					
	Sourness	Sweetness	Bitterness	Saltiness	Umami	Astringency
1	1.00	1.50	1.40×10^{-3}	1.00	0.20	0.10
5	4.00	3.00	2.80×10^{-3}	2.00	4.00	0.50
9	8.00	6.00	1.40×10^{-2}	10.00	20.00	1.00

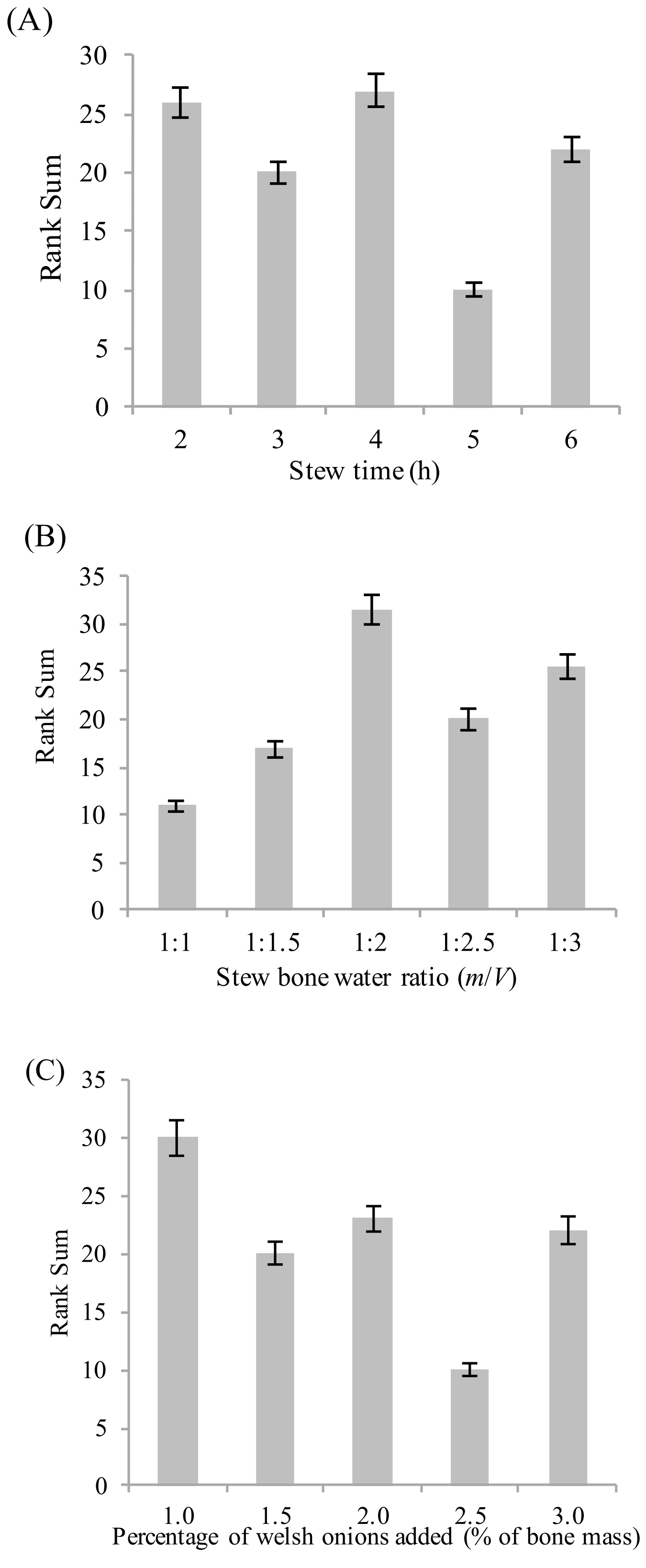

Figure S1. Optimization of stewing conditions: (A) Stew time; (B) stew bone–water ratio (m/V); and (C) ratio of welsh onions added (percentage of bone mass) by sensory evaluation.
